# Emerging Strategies in 3D Culture Models for Hematological Cancers

**DOI:** 10.1097/HS9.0000000000000932

**Published:** 2023-07-27

**Authors:** Dafne Barozzi, Cristina Scielzo

**Affiliations:** 1 Università degli Studi di Milano-Bicocca, School of Medicine and Surgery, PhD program in Molecular and Translational Medicine (DIMET), Milano, Italy; 2 Unit of Malignant B cells biology and 3D modelling, Division of Experimental Oncology, IRCCS Ospedale San Raffaele, Milano, Italy

## Abstract

In vitro cell cultures are fundamental and necessary tools in cancer research and personalized drug discovery. Currently, most cells are cultured using two-dimensional (2D) methods, and drug testing is mainly performed in animal models. However, new and improved methods that implement three-dimensional (3D) cell-culturing techniques provide compelling evidence that more advanced experiments can be performed, yielding valuable new insights. In 3D cell-culture experiments, the cell environment can be manipulated to mimic the complexity and dynamicity of the human tissue microenvironment, possibly leading to more accurate representations of cell-to-cell interactions, tumor biology, and predictions of drug response. The 3D cell cultures can also potentially provide alternative ways to study hematological cancers and are expected to eventually bridge the gap between 2D cell culture and animal models. The present review provides an overview of the complexity of the lymphoid microenvironment and a summary of the currently used 3D models that aim at recreating it for hematological cancer research. We here dissect the differences and challenges between, and potential advantages of, different culture methods and present our vision of the most promising future strategies in the hematological field.

## INTRODUCTION

Hematological malignancies are categorized by site, depending on where the cancer is first detected among blood, bone marrow (BM), or secondary lymphoid organs (SLOs). The niches in which malignant cells arise and home provide a supportive and protective microenvironment, which contributes to tumor development and drug resistance. Those niches present complex internal architectural, cellular, and molecular-defined microenvironments that influence cell behavior in both physiological and pathological conditions.^1^

In recent decades, there has been a growing awareness of the dissimilarity between flat cultures and the complex extracellular microenvironment in which cells live in physiological conditions. The internal complexity of the tumor microenvironment (TME), coupled with the heterogeneity introduced by malignant cells, implies that conventional two-dimensional (2D) cultures are no longer efficient models to reproduce the pathology in vitro. In the same way, animal models present multiple limitations because they do not often properly reproduce the features of human pathobiology besides being expensive and time consuming.^1,2^

Three-dimensional (3D) in vitro tissue modeling could be the right compromise between conventional 2D cultures and animal models. Several studies highlighted the many differences between 2D and 3D cell cultures demonstrating that cells cultured in a more complex microenvironment resemble their in vivo counterparts in morphology, interactions with other cells and extracellular matrix (ECM), gene and protein expression levels, sensitivity to external factors, proliferation and differentiation status.^3,4^

Thus, the development and validation of 3D methods to overcome these limitations is a field in active expansion, especially with regard to solid tumors.^1,5^ Due to the nonadherent nature of blood cancer cells, the application of the 3D approach in hematological fields is particularly challenging for researchers. However, scaffold-based models mimicking the specialized microenvironment of lymphoid tissues are recently gaining ground, integrating advanced biomaterials and micro/millifluidics with the potential to recreate the dynamic nature of these diseases.

The focus of this review is to give an overview of the state of the art about 3D models currently in use to investigate different hematological malignancies, with a particular attention to modeling the TME in both static and dynamic systems.

## 2D VERSUS 3D CULTURE: IN WHAT THEY DIFFER

Two-dimensional cultures have been traditionally considered the gold standard in vitro model for cell biology, being cheap, easy to handle, time saving, and reproducible. However, 2D cultures cannot be exploited to reproduce a complex functional microenvironment composed by multiple cell types. In vivo, cells live and carry out their functions into an intricate ECM network enriched with stromal cells, immune cells, blood vessels, and cell-secreted factors. Several studies demonstrated that cells cultured in monolayers had a different behavior if compared with cells grown into a 3D context.^3,4^ The first striking difference concerns cell morphology: as the term flat cultures suggests, cells in 2D assume a planar morphology, independently from their natural being. For example, mesenchymal stem cells (MSC), largely employed to reconstruct the BM niche in vitro, are usually defined as spindle-shaped cells, indicating their alter ego in monolayer cultures, whereas MSC grown in 3D structures extend in all directions assuming a stellate morphology.^6^ Cellular shape is controlled by the distribution of the adhesive pattern in the microenvironment, as well as integrin distribution, cell survival, and the expressions of specific gene pathways. It is now evident that all cells have perceptions of the ECM topography, rigidity, and anisotropy, and behave differently depending on microenvironment variations—modifying their morphologies, cytoskeletal organizations, and adhesion sites.^4,7^ The most involved players in these scenarios are as follows: (1) integrin receptors, which form multiprotein adhesion complexes that take part in the sensory system of cells that respond to biochemical and mechanical stimuli; (2) focal adhesions, which act as a link between the cellular actin cytoskeleton and the ECM via integrin receptors, developing only in the presence of active loads on the actin network, whose modifications drastically change the adhesion pattern of the cells.^7^

In tissues in vivo, mechanical forces are conveyed to the cells dependent on the ECM fibrillar architecture in which they are embedded;^4,8^ moreover, immune cells face numerous solicitations as they circulate in the bloodstream, encountering various tissue types.^8^ These considerations highlight another significant difference between 2D and 3D cultures. Indeed, in the monolayer cultures, cells do not undergo mechanical stimulations, so are unlikely to represent in vivo mechanotransduction mechanisms. On the contrary, 3D cultures allow for maintenance of the spatial distribution of adhesion complexes, and of the morphology and interaction of cells with different cell types and ECM, reflecting the physiological cell function and signaling. To date most of these mechanisms are unknown, although there is evidence to support this statement, for example regarding cell differentiation. The 3D network seems to better stimulate cell differentiation and the stabilization of terminally differentiated cells. Suresh et al compared 2D and 3D cultured adipose-derived stem cells (ADSCs) stimulated with proangiogenic media. The effect of those factors was enhanced by the presence of a 3D structure, which produced a higher expression of CD31 and the capacity to form tubular-like structures in Matrigel cultures.^9^ Fischer et al also found that the presence of a dynamic condition and the forces related to it, coupled with the presence of proper medium conditions, further stimulates the expression of CD31 in CD31-negative ADSC.^10^

**Figure 1.**
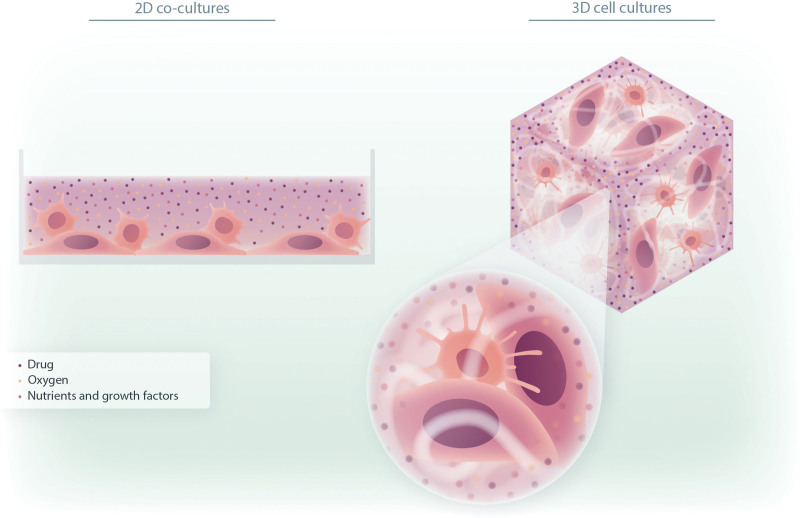
**Schematic representation of the comparison between 2D and 3D cell co-cultures.** The 2D co-cultures include the adhesion of cells to a planar surface, forcing them to assume a flat morphology. Cells growing in adhesion cover the entire plate; floating cells generally adhere on their top. This homogeneous distribution gives cells access to the same supply of oxygen and nutrients, as well as of drugs, only from above. The 3D cell cocultures provide a multidimensional structure in which cells can interact and produce an ECM network, generating a complex environment. This organization more closely mimics the TME, also enabling extension of the study of circulating malignant cells relative to their behaviors in a tissue-like environment. This complexity provides a different accessibility to the soluble factors that can fully interact with the different microenvironment components (cell types), enabling a more efficient study of therapeutic effects related to the presence of a TME. ECM = extracellular matrix; TME = tumor microenvironment; 2D = two dimensional; 3D = three dimensional.

Less is known about non-adherent circulating cell types (eg, leukemic cells). Recently, we established a long-term bioprinted 3D culture model of chronic lymphocytic leukemia (CLL), in which we demonstrated that a 3D architecture enhances the viability of CLL primary cells over time. To understand the survival differences and advantages reached in 3D culture, we performed an RNAseq analysis to compare 3D bioprinted CLL versus 2D cultured cells. Interestingly, we identified different modulations of a variety of genes; for example, we observed upregulation of the *CXCR3* gene, involved in leukocyte trafficking, integrin activation, and cytoskeletal and focal adhesion remodeling (ie, the *HCLS1* gene, involved in the migration and homing of CLL cells^11^ and prognostic factors^12,13^). These results also suggest that, for cells usually grown in suspension in vitro, we can obtain a different phenotype in 3D culture; this offers the possibility of recreating the spatial organization and disposition found in tissues in vivo^14,15^ (Figure 1). Another important aspect to consider is that cells arranged in monolayers or in suspension receive all the same amounts of oxygen, nutrients, and growth factors, and consequently more cells will be in the same phase of the cell cycle. In 3D cultures, cells are heterogeneously exposed to oxygen and medium, resembling the situation occurring in tissues or tumoral bulks, thus possibly overcoming this limitation.^3^ Indeed, tumoral spheroids in static conditions can reproduce the inactive and hypoxic core that characterizes solid cancers.^16^ These statements are directly linked to the field of drug screening and related issues; around 90% of drugs screened in preclinical models do not match with subsequent human clinical trials, meaning a huge economic loss but also a waste of time and resources.^17^ In vitro, the accessibility of cells to drugs depends on their different disposition in the culture plate and on the penetration ability of the different compounds (Figure 1). Moreover, the lack of a microenvironmental neighborhood causes the absence of a protection context against drugs or otherwise even a co-adjuvant effect to drugs, implying an entirely different behavior of cells toward pharmacological treatments, that needs to be investigated.^16^

Despite all the advantages arising from the use of 3D models in biomedical research compared with 2D models, there are crucial aspects that must be implemented, such as the standardization and reproducibility of the protocols for readout analysis. These aspects are summarized in Table 1.

**Table 1:**
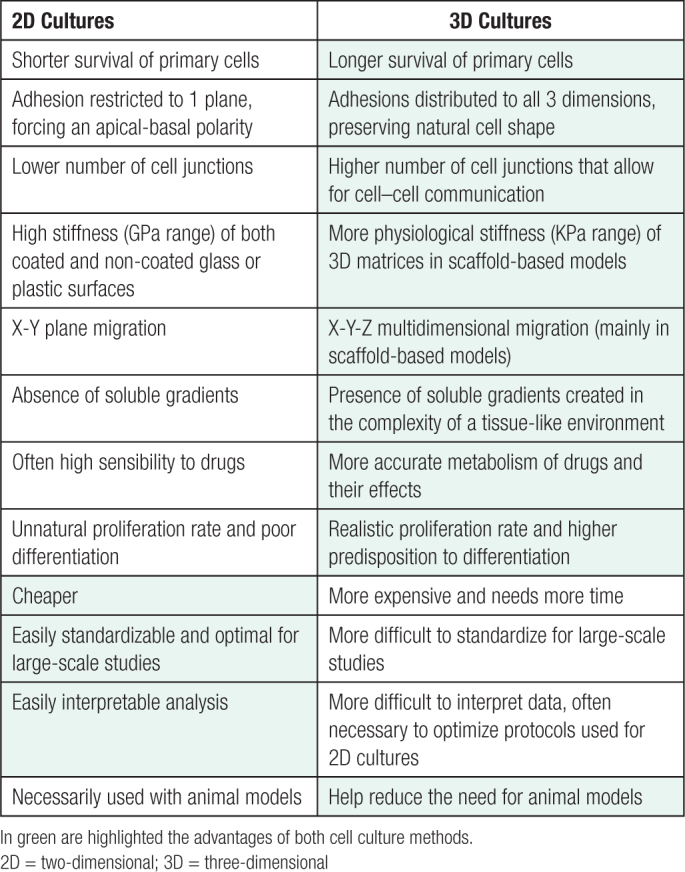
Advantages and Disadvantages for 2D and 3D Cultures are Reported

The 3D in vitro models have another great potential in helping reduce the use of animals as preclinical models. This is an advantage in different ways, from addressing the ethical issues to the benefit of disposing with human-derived models. To date, 3D models cannot replace animal models for many reasons, one of which is that those models have often lacked the complexity of human organs (eg, vascularization). However, the biomedical research direction follows the 3Rs principle (replacement, reduction, and refinement), to minimize the use of animal models when valid alternatives are available.^18^

## THE COMPLEX LYMPHOID MICROENVIRONMENT IN HEMATOLOGICAL CANCERS

In contrast to solid cancers, which usually require genetic modification and cellular reprogramming to metastasize and disseminate, leukemic cells retain an innate ability to migrate into different tissues, and to survive in the circulatory system while also acquiring the potential for uncontrolled division. For this reason, it is important to consider that hematological cancers can be found in many anatomical sites in the human body.^19^ There has recently been a growing interest in the use of 3D systems for hematological malignancies. In the field of blood cancers, 3D modeling is evolving, with the aim of reconstructing the lymphoid microenvironment and its interactions with neoplastic cells. The tissue microenvironment is a critical element of hematological tumors. For example, in CLL, the active neoplastic cells are those accumulating in tissues, indicating a crucial role of the microenvironment in the progression of the neoplasia.^2,11,20^ Another important outcome for non-solid tumors research is about the understanding of the mechanisms underlying dissemination of malignant cells through the bloodstream, and their ability to home in tissues.^19^ For this reason, the use of dynamic 3D cultures to enable the recirculation of malignant cells within the system is becoming more and more popular, mimicking the blood flux and the forces related to it and to different tissues that will be discussed later in this review.

In general, blood cancers are extremely heterogeneous diseases with often unprecise natures, various patterns of clinical presentation, and responses to treatments; therefore, it is particularly complex to accurately characterize them.^21^ This inter and intratumor heterogeneity, which involves different microenvironments, reflects the need for a better understanding of the pathobiology, necessarily exploiting systems in which neoplastic cells can survive and behave as they do in vivo.^1^ Given the influence of the microenvironment on cell behavior, ranging from soluble signals to mechanical stimuli, to develop such systems, it will be essential not only to implement a 3D culture but also to include additional external components.

**Figure 2.**
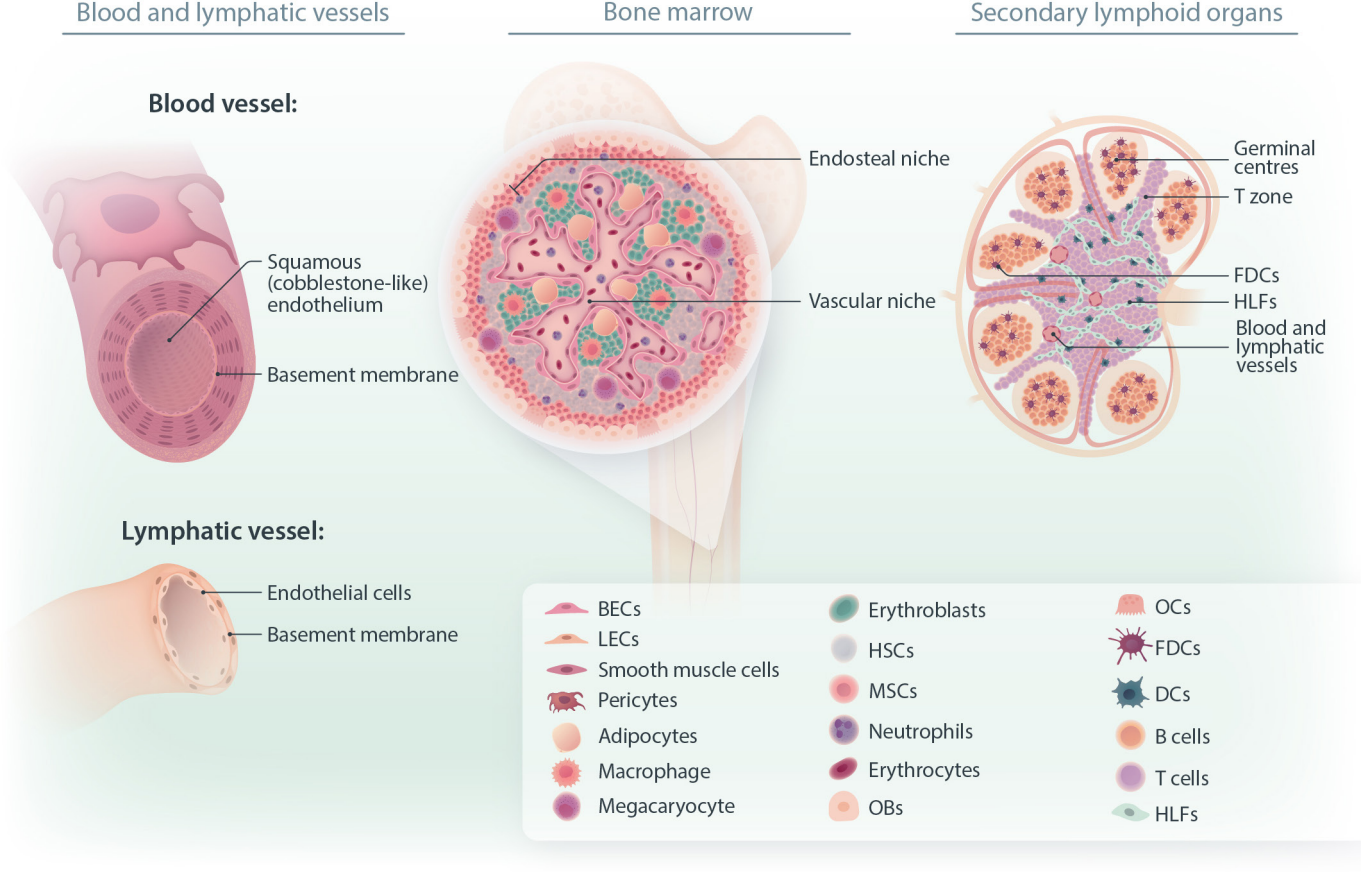
**This illustration shows the architecture and composition of different lymphoid microenvironments mainly interested by different blood cancers.** Lymphatic and blood vasculature are of main interest in modeling the hematopoietic microenvironment, blood and lymph flow, and interactions between endothelial and malignant cells. Primary and secondary lymphoid organs are the main districts in which hematopoietic malignancies arise or do homing during their journey circulating into the vessels network. Here, we give an overview of the main components and functional structures of these tissues.

Herein are summarized the main components of the lymphoid microenvironments that current and future studies should consider building a functional microenvironment in vitro (Figure 2).

### Blood and lymphatic vessels

Blood and lymphatic vessels generate an intricate and organized network, used by malignant cells as a route for metastatic dissemination. Although an excess of malignant circulating white blood cells in the peripheral circulation is a characteristic feature of leukemias, this scenario may also be associated with other blood cancers such as lymphomas or multiple myeloma. The importance of in vitro vascular modeling relates to the vascularization of the 3D scaffold, to facilitate the nutrient and oxygen supplies to the entire structure, and to provide a necessary in vivo-like interactive surface for neoplastic cells. Leukemic cells extravasate to enter hematopoietic organs (as do healthy leukocytes), exploiting vasculature interactions to transmigrate toward specific supportive niches such as BM or SLO. After that, colonizing cells can stimulate upregulation of adhesion molecules by the endothelium (eg, E-selectin),^19^ encouraging the homing of other neoplastic cells. Leukemic cells are present, both as resident cells in tissue niches and as circulating cells in the bloodstream, entering and exiting niches in a loop of extra and intravasation that is essential for disease progression.^19,22^ A growing interest in modeling vascularized microenvironments has emerged, to study the trafficking of immune cells. Kotha et al. engineered a human vascular marrow niche to study the interactions directing hematopoietic stem progenitor cell (HSPC) trafficking, demonstrating the possibility of studying the dynamic spatial and temporal interactions between HSPC and different microenvironment components.^23^ The presence of vasculature in 3D models of hematopoietic neoplasia is also involved in the potential for these models to be used in drug screening, significantly impacting outcomes. For example, one of the approved drugs for CLL treatment is ibrutinib, an inhibitor of Bruton tyrosine kinase (BTK), antagonizing the B-cell receptor (BCR) signaling pathway. This inhibition of BCR activation hinders the triggering of VLA-4-dependent adhesion of CLL cells to ECM and to endothelium, causing mobilization of neoplastic cells in the bloodstream and interfering with the microenvironment’s protective effects.^24^

Diving into the tissue model that we need to recapitulate, it is necessary to remember that blood and lymphatic vasculature are organized in a hierarchical structure (Figure 2). Blood vessel networks start with the aorta, which branches into gradually smaller vessels until the capillaries. Lymphatic vessels, by contrast, start with blind-ended capillaries, which collect and transport excess interstitial fluid into larger vessels until reaching the thoracic duct, which empties into the inferior vena cava, returning the fluids into the bloodstream.^25,26^ The different branches of both networks present different diameters, wall thicknesses, cell types, ECM compositions, and other different functions. For example, lymphatic capillaries lack a basement membrane and are lined by a single layer of overlapped lymphatic endothelial cells (ECs); this lack of intercellular junctions allows for the uptake of fluid, macromolecules, and cells.^25^ Blood vessels are composed of elastin, collagen, proteoglycans, and blood endothelial cells (BECs). Their walls can be divided in to 3 main layers: generally, the inner stratum is composed of a layer of BECs, the middle of aligned smooth muscle cells, elastin, and collagen, and the outer comprises fibroblasts, elastin, and collagen. The composition of these compartments varies across the different offshoots, conferring specific mechanical features for blood circulation to them.^26^ This introduces another important aspect to consider when approaching the modeling of blood cancers, because circulating cells in vivo undergo mechanical forces generated in the dynamic vascular flow, as blood pressure and fluid shear stress. Vascular 3D in vitro models also offer the ability to mimic the stresses imposed on circulating cells in the bloodstream, adding an important stimulus implied in neoplastic cell behavior.^26,27^

### Bone marrow

The BM microenvironment is our natural blood factory, which serves as a stem cell niche, ensuring an effective hematopoiesis, balancing the preservation of a long-term self-renewing hematopoietic stem cell population, with the production of mature blood and immune cells.^1,28^ In physiological conditions, the BM microenvironment is organized in spatially defined niches: endosteal and vascular (Figure 2). The former counts for <10% of the total BM volume and comprises MSC, osteoblasts (OB), osteocytes, osteoclasts (OC), and a small percentage of HSPC.^1^ The main role of the endosteal niche is promotion of HSPC quiescence, preserving a self-renewing population. The vascular niche hosts the majority of HSPC of the BM and can be further subdivided into arteriolar and sinusoidal niches. Generally, the hematopoietic microenvironment is hypoxic, with a presence of reactive oxygen species around the sinusoids, stimulating migration and differentiation of HSPC. Instead, the arterioles reside bordering the endosteal niche presenting less permeable walls and higher oxygen concentrations to maintain HSPC quiescence. The vascular niche is composed and regulated by a wide variety of cell types such as endothelial cells (EC), different populations of MSC, adipocytes, and HSPC at different maturation stages.^1^ ECM also plays an essential role in the BM microenvironment, in activation, retention, and quiescence of HSPC through interactions with adhesion molecules (eg, integrins or selectins). An important stimulus given by the matrix is dependent on its stiffness; HSPC rearrange their cytoskeletons depending on the substrate on which they are bound, and this mechanical stimulus is converted into a specific biochemical response, influencing cell behavior.^29^ The BM matrix can be divided into inorganic (ie, hydroxyapatite) and organic components, the latter constituted by collagenous (eg, collagen IV) and noncollagenous proteins such as fibronectin, elastin, hyaluronic acid, and osteopontin.^1,29^

The need to maintain in vitro cultures of self-renewing HSPC for different purposes, from differentiation studies to clinical need, is increasing interest in engineering an in vitro BM niche model.^28^ This is also true for hematological malignancies. Alterations in the state of BM homeostasis leads to exhaustion of the production machinery, affecting the maintenance of HSPC stemness and/or differentiation potential. In hematological malignancies, the BM microenvironment is often involved; neoplastic cells compete with normal HSPC for space and survival, disrupting normal hematopoiesis, occupying and educating the stromal niche to be supportive and protective toward the tumor itself.^1^ In leukemias, for example, the BM results in overload by malignant cells, a small proportion of which regain stem cell properties and rearrange the microenvironment into a shelter for their own proliferation and protection.^29^ Another important neoplasia that is significantly involved in the BM niche is multiple myeloma (MM), in which the tissue is infiltrated by malignant plasma cells, associated with high levels of monoclonal proteins. In the BM niche, MM cells adhering to ECM and MSC upregulate a wide variety of factors (eg, IL6 and SDF1α) that favor the growth, survival, migration, and drug resistance of malignant cells.^30^ In general, during the progression of hematopoietic cancers, the BM microenvironment undergoes a process of remodeling in which angiogenesis plays a pivotal role, promoting the supply of oxygen, nutrients, and soluble factors.^1,30^

Thus, the BM niche is a complex microenvironment of great interest to 3D in vitro modeling, both for blood cancer pathophysiology studies and for uncovering tumors’ mechanisms of resistance to treatments.

### Secondary lymphoid organs

T and B cells traveling through our body scan for related antigens in the SLO that coordinate immune responses, providing an optimal microenvironment for immune-cell activation.^31^ SLO are highly complex and dense structures characterized by a defined cell compartmentalization that is essential to their functions.^31,32^ The different peripheral lymphoid organs (ie, lymph nodes [LN], spleen, tonsils, and mucosal-associated lymphoid tissues) are all similarly designed to collect local or systemic antigens. The architecture is characterized by a network of stromal cells that differ between the tissues, blood, and lymphatic endothelial structures, and B and T cell zones^33^ (Figure 2).

LN are located at vascular junctions and are served by several lymphatic and blood vessels. Internally recognized a paracortical region called T zone can be identified, in which follicles of densely packed naïve B cells arise and, once activated, proliferate developing germinal centers. Follicular dendritic cells are also found inside the follicles, and are antigen-presenting cells important to maintaining the follicles structure.^31,33^ Antigens and cells enter the LN via afferent lymphatic vessels, filtered through the lymphatic sinuses in the medulla, and transported into the LN cortex, before leaving through efferent lymphatic vessels.^33^ Another means of entry for naïve B and T lymphocytes are the specific blood endothelial structures called high endothelial venules (HEV), found in the paracortical T cell area.^31^

The structural backbone of LN consists of an intricate network of stromal reticular cells (fibroblastic reticular cells [FRC]) composed mainly of human lymphatic fibroblast (HLF), which produce and organize a complex meshwork of fibrillar proteins such as collagen VI.^31,32,34^ The FRC network exploits an essential role not only in the architectural organization of the organ, but also in acting as a conduit for the rapid delivery of antigens and chemokines, and for lymphocyte migration throughout the LN parenchyma.^32^ FRC cooperate with HEV and lymphatic endothelium in directing the migration of cells to their specific compartment through the production of cytokines and expression of adhesion molecules (eg, VCAM-1 and ICAM-1). T and dendritic cells are attracted to the paracortical region by their expression of CCR7 (receptor for CCL19 and CCL21), while B cells expressing CXCR5 are chemoattracted to follicles by CXCL13.^32,33^

SLO are stations for circulating immune cells and are often involved in hematological neoplasia initiation, proliferation, and/or spreading. Leukemias, being typically widespread diseases, often disseminate in SLO exploiting normal immune-cell trafficking.^19^ Modeling of SLO is of primary importance for malignancies arising from these structures as lymphomas, a highly heterogeneous category of blood cancers that can be primarily divided into Hodgkin and non-Hodgkin (NHL) diseases. NHL is further divided into B- and T-cell lymphomas (B-NH, T-NH). The categorization of lymphomas is extremely diverse with a wide range of variables, most often arises from LN or other SLO, and some subtypes also frequently disseminate in extranodal sites.^21^ The interest in reproducing in vitro SLO (in particular LN) is high, because such an accomplishment would offer a platform not only for recapitulation of hematological malignancies and their dissemination, but also for the study of their healthy counterparts, to dissect the molecular and biophysical mechanisms at the base of the immune response.

## MODELING THE LYMPHOID MICROENVIRONMENT IN 3D FOR BLOOD CANCERS: STATE OF THE ART

**Figure 3.**
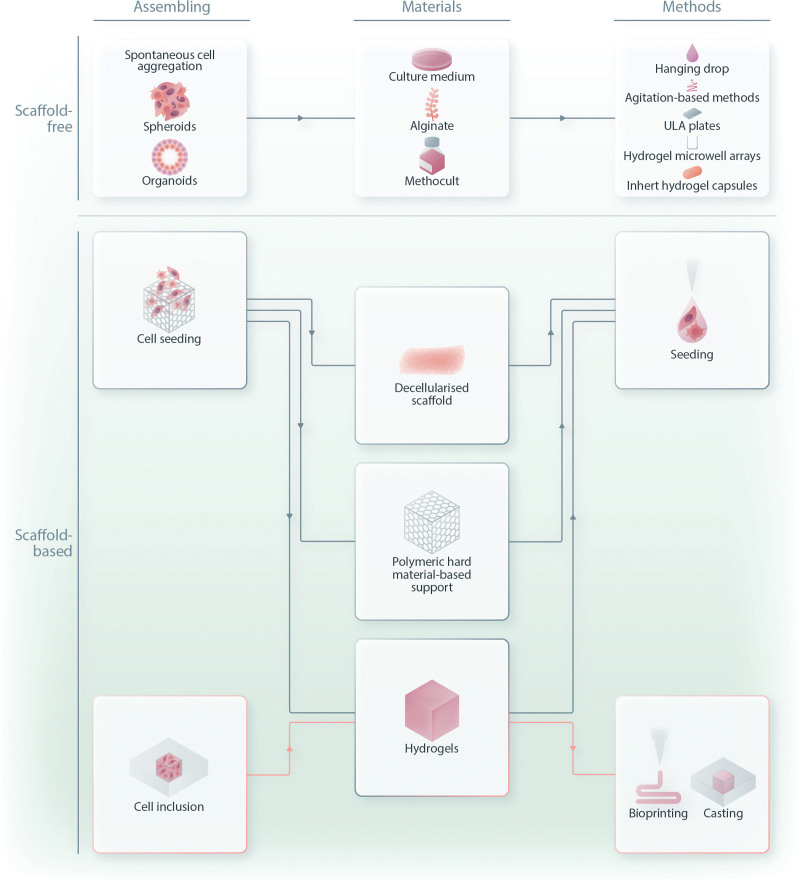
**This diagram aims to provide a clarified subdivision and nomenclature of the different 3D culturing techniques used to model hematological cancers in 3D.** In this review, we categorized these methods as scaffold-free and scaffold-based. Scaffold-free techniques include a wide variety of standardized platforms, materials, and procedures. These methods aim to produce self-assembled structures as spheroids and organoids, which, particularly for hematological neoplasm 3D modeling, may need hydrogel-coated platforms or external capsules to guarantee stability. Scaffold-based methods have been subdivided according to the approach (seeding or inclusion) and, consequently, on materials used. This approach differs from the one described above, because here cells lay and organize within a matrix mesh that act as a structural, and eventually functional, support for cellular growth, differentiation, and ECM network production. ECM = extracellular matrix; 2D = two dimensional; 3D = three dimensional.

In the field of 3D culture, there is still a lot of confusion about nomenclature; often, different names refer to the same approach. We will follow the classification that we think is the most representative, which subdivides 3D culture methodologies into 2 major groups: scaffold-free and scaffold-based methods (Figure 3).

As the name suggests, scaffold-free models include various techniques based on the formation of cellular aggregates without the use of an external matrix that interacts with the cells. Scaffold-based models are based on the inclusion or seeding of cells into a scaffold that acts as a biophysical support for cellular growth and aggregation. In the hematological malignancies scenario, due to the floating nature of the cells, these 2 categories overlap. We will describe scaffold-based methods, such as technical methods or platforms in which cells are placed within a matrix network that actively supports cell growth, proliferation, and biological activities, both mechanically and biologically. This should not be confused with the methods that involve the use of hydrogels in scaffold-free models. In this case, the matrix is used to support the 3D sample, only to keep it intact and stable by avoiding disruption during culture manipulations.

Table 2 shows a schematic classification of the different 3D cell culture methods currently used to model blood cancers in vitro.

**Table 2:**
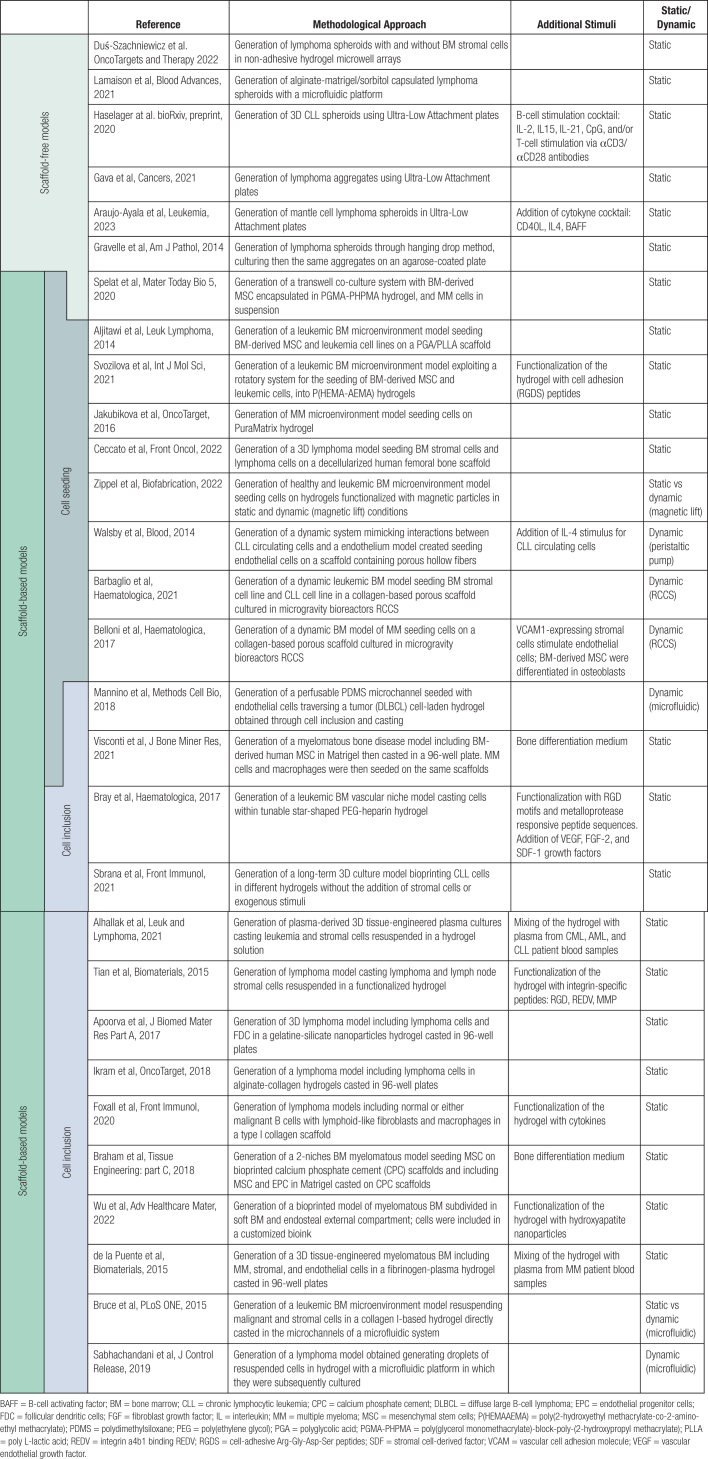
A Summary of the State of the Art on 3D Modeling for Blood Cancers Subdivided for Techniques and Culturing Method Features

### Scaffold-free models

The principle behind scaffold-free techniques is the generation of cell aggregates (ie, spheroids and organoids) using specific platforms that can exploit gravity, such as the hanging drop method.^35^ There are also agitation-based methods, ultra-low attachment-coated plates,^14^ or inert hydrogel capsules.^3^ Those kinds of platforms are optimal for multiple reproducible replications (which is very useful for drug screening analyses) and are generally easy to handle. In an interesting work, Haselager et al set up a model mimicking in vivo CLL-T cell stimulation by generating spheroids with PBMC from CLL patients. They obtained a scalable platform able to sustain primary CLL cell growth and simulate an in vivo-like microenvironment stimulation, which could be useful in large-scale studies as a drug screening method.^14^ Recently, Araujo-Ayala et al generated a 3D spheroid model of LN TME of mantle cell lymphoma (MCL), which recapitulates in vivo responses to target therapies. They demonstrate that this model fairly represents fundamental hallmarks of MCL microenvironment features as proliferation signature, BCR signaling pathway, and immune TME exhaustion.^36^

A limitation of spheroid models is poor diffusion of oxygen and nutrients in the inner core of the structure, which depends on the size of the sample. In addition, spheroids are difficult to handle outside the seeding platform and tend to self-disassemble, especially when using nonadherent cells as lymphocytes.^1,3,37^ The creation of a co-culture adding a stromal component is an efficient solution to obtain a more compact and cohesive spheroid.^38,39^ Duś-Szachniewicz et al, exploiting a non-adhesive agarose microwell system, developed a 3D model of hybrid spheroids to study the crosstalk between B-cell non-Hodgkin lymphoma and MSC cells. The presence of a stromal population enabled the manipulation and processing of spheroids for histological analyses, because stromal cells are able to produce ECM networks that can both physically trap and biologically attract non-adherent cells.^1,38,40^ Furthermore, the stromal component added a higher level of complexity to the model, mimicking the crosstalk that takes place within the microenvironment. Another approach that could be exploited for the generation of more compact models, besides adding ECM-producing cells to the culture, is encapsulation of self-assembling spheroids in hydrogel shells to trap the aggregates in a defined space.^41,42^ In this context, Lamaison et al generated high-throughput follicular lymphoma and diffuse large B-cell lymphoma (DLBCL) spheroids, using a highly reproducible microfluidic method based on the encapsulation of cells inside permeable, hollow alginate microspheres.^41^ Both models were used to perform drug screening analyses on multiple samples. Results of such analyses confirmed findings commonly observed in 3D solid tumor models: the 3D microenvironment tends to physically and biologically protect neoplastic cells against drugs, getting closer to the in vivo condition. An interesting model, which can be considered a hybrid between scaffold-free and scaffold-based, has been developed by Spelat et al, who wanted to integrate an MM culture and a 3D microenvironment while maintaining malignant cells in their natural floating condition.^42^ They observed that this interaction led to a different MM cell behavior when compared with 2D cultured cells, highlighting an overexpression of CX3CR1, involved in the axis CX3CL1/CX3CR1, which supports cooperation between malignant cells and the microenvironment.^42^

Great advances have been made in the 3D modeling of nonsolid tumors; there are currently several improvements that can be made to facilitate the creation of these models, such as the use of scaffold matrices and dynamic systems, which is discussed in a subsequent section in this review.

### Scaffold-based models

Reproduction of a tissue microenvironment requires the structural and compact organization of different cell types that can accommodate, in our case, malignant cells of hematopoietic origin. As we already pointed out, hematological tumor cells interact with various components of the microenvironment, such as endothelium, stromal cells, ECM, and soluble factors. The building of such a complex model can be enhanced by the employment of scaffold matrices, which literally support the cells and can help both in architectural organization and biological activities.^3,37^ The presence of such a support could potentially be a strategic advantage for 3D cultures, first of all by being of great technical help in the seeding, maintenance, and handling of the sample. Scaffold-based 3D modeling enables different matrix conformations and (according to the specific objectives), the best options in terms of shape, internal architecture, and mechanical properties can be selected.^37^ For example, Svozilová et al optimized a BM niche model of leukemia, recapitulating the trabecular bone structure using a hydrogel scaffold, using needle-shaped ammonium oxalate as a porogen.^43^ Apoorva et al designed biomaterials-based hydrogels with tunable mechanical stiffness for B lymphoma growth, demonstrating that a lymphoid-like stiffness influences BCR expression levels in malignant cells, with consequences for proliferation and drug resistance.^44^ Along with the possibility of tuning physical properties, matrices can also be functionalized with a wide variety of compounds to make them biologically active.^45,46^ In a remarkable work, Bray et al generated a 3D triculture that mimics the interactions between acute lymphoblastic leukemia (ALL) cells and the vascular niche. They used a tunable star-shaped polyethilene glycole (PEG)-heparin hydrogel functionalized with RGD motifs to enhance cellular adhesion, and metalloprotease responsive peptide sequences to enable localized cellular remodeling. In this model, they used MSC, human umbilical vein endothelial cells, and leukemic cells, demonstrating the potential to create an environment able to mimic the mechanisms of drug resistance (ie, adhesion-mediated pathways). The presence of a scaffold enabled it to enhance the complexity of the microenvironment, as it physically sustains the formation of vascular structures and recapitulates the mechanical features of tissues.^47^

Shifting attention from materials to methods, 2 main techniques were exploited for the development of 3D scaffold-based models: cell seeding on a preformed scaffold and cell inclusion in hydrogels. Seeding of cells on a matrix enables employment of different types of materials, from polymeric porous hard materials to hydrogels, and also decellularized human scaffolds.^48^ Polymeric hard scaffolds can be synthetic (eg, poly L-lactic acid [PLLA] and polyglycolic acid [PGA])^49^ or made of natural materials.^37^ One of the most common natural hard matrices used is based on the collagen fibers. Recently, we generated a 3D culture model for CLL and MM BM microenvironment, exploiting a collagen-based porous hard scaffold.^20,50^ The presence of collagen in the support facilitated cells entering the matrix, and the complex fibrous network enhanced cellular organization and cell–cell interactions. Polymeric hard materials are an optimal solution for modeling stiffer tissues (eg, BM),^20,50^ whereas hydrogels are reticulated structures of crosslinked polymer chains that possess high water content and the ability to simulate soft tissues.^37,51^ Similarly to natural hard scaffolds, hydrogels of natural composition, typically consisting of either protein and ECM components (eg, collagen, fibrin), or of other biological sources (eg, alginate), are biocompatible and bioactive and able to promote several cellular functions, such as proliferation or viability.^44,51^ Synthetic hydrogels (eg, polyethilene glycol diacrylate [PEGDA], polyethilene glycole [PEG], polydiglycerol adipate [PDGA], PuraMatrix)^52,53^ are biocompatible materials; in contrast, natural hydrogels are not bioactive. Furthermore, nonnatural hydrogels are more resistant and stable, and are more easily reproducible in their composition.^51^

Another technique for 3D scaffold modeling involves inclusion of cells in a hydrogel matrix. Liquid hydrogels are crosslinked to achieve specifically shaped structures that can be obtained by casting the suspension into a mold or through bioprinting.^5^ Casting of cell-enriched hydrogels can be performed exploiting multiwell plates or custom-made molds (eg, 3D printed plastic molds).^54^ Another possible approach that could be exploited is the concomitant use of techniques typically employed for scaffold-free modeling, such as hanging drop or reverse hanging drop, in combination with hydrogel-based scaffolds—a technique that we will call manual droplet casting.^44,53,55,56^ Foxall et al developed a 3D model of DLBCL TME, exploiting a type I collagen-based matrix mixed with a cytokine-enriched medium, obtaining scaffold-based spheroids by the hanging drop method.^56^ Differently from scaffold-free spheroids, those cultures remain mixed with the hydrogel, which can be used as a structural and functional support for their development. Finally, there is the bioprinting strategy, an additive manufacturing technique, which incorporates bioinks that are constituted of cells resuspended in biocompatible materials.^57^

Seeded scaffolds enable culture of cells in 3D without stressing them and giving them complete freedom to organize, but have 2 main limits: the technical variability produced by multiple seedings, and difficulty in obtaining a specific, customized, and precise architecture and compartmentalization. The 3D bioprinting has a great versatility and it allows the generation of multiple scaffolds with high levels of reproducibility, enabling precise control of scaffold composition, spatial distribution of cells and matrix, and specific architecture of the structure.^57^ In literature, there are multiple examples showing the potentialities of this technique.^58,59^ Wu et al fabricated a model of MM BM niche through bioprinting. They used a customized bioink and coaxial bioprinting technology to obtain a complex layered scaffold that includes a central core mimicking the soft BM and an external sheath mimicking the cortical bone.^58^ As previously mentioned in this review, we recently published a work on CLL 3D bioprinted models, demonstrating the ability of the technique to maintain viable cells (eg, patient-derived cells) in long-term 3D cultures. The work also demonstrated the advantages of 3D modeling, proving that the third dimension, even without the addition of ECM and stromal cells, overturns the behavior of CLL cells, and suggests that 3D cultures resemble a more physiological microenvironment.^11^

## ADVANTAGES OF DYNAMIC CULTURES

**Figure 4.**
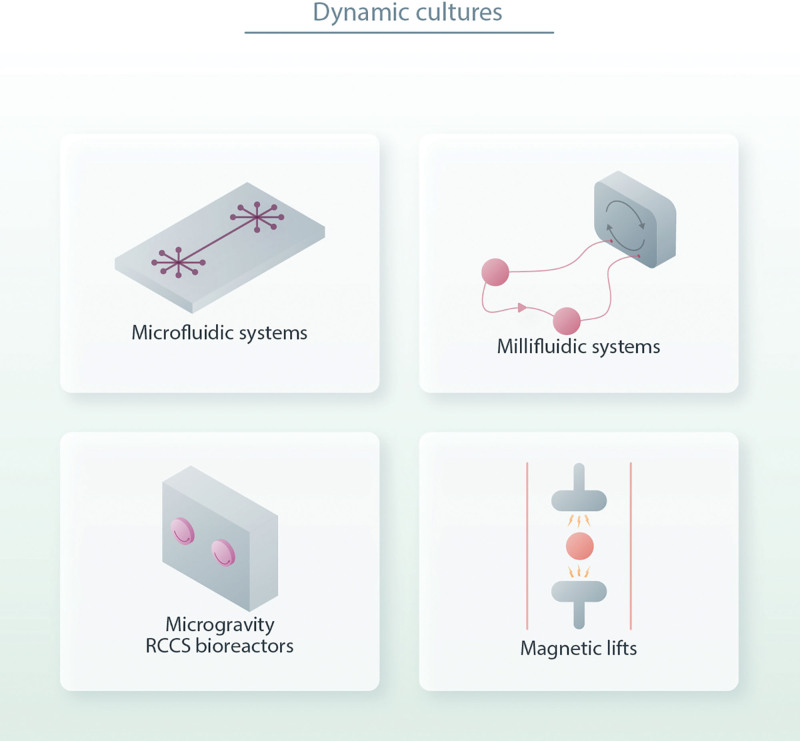
**Schematic representation of platforms for dynamic cultures considered in this review.** Because of the need to add a vascular component in the 3D cultures, and the necessity of studying mechanisms of dissemination and homing of neoplastic cells, it has become crucial to add a dynamic component to the generation of these models. The dynamic stimulation can be induced through a (micro- or macro-) fluidic system that allows for the recirculation of soluble factors and cells investing the 3D structure. Other approaches include agitation-based bioreactors exploiting microgravity (RCCS) or magnetic lifting. 3D = three dimensional.

Up to this point in this review, we reported the possible advantages of using 3D culture in vitro models for controllable analysis of hematological cancer behavior and advancement in preclinical testing. Nevertheless, these methods are still far from in vivo conditions, as they lack some critical features; among them are controlled nutrient supply, waste removal, and biophysical forces such as pressure and shear stress. Indeed, continual improvement in the 3D culture field leads toward microfluidic and macroscopic systems of increasing complexity (Figure 4). These systems enable the capture of multiple features of cell–cell and cell–ECM interactions in a 3D environment, while tightly monitoring perfusion in real time and offering a unique system for carefully resembling cancer cell interactions, especially with the immune system.^60^ These platforms represent a good compromise between conventional 2D cultures and animal models, because they can be exploited to perform relevant reductionist studies on cancer cells interacting with the microenvironment, and they can be easily adapted according to the experimental requirements.^61^

An example of a microfluidic model of hematological cancer is the generation of 3D tumor-stromal-immune cell spheroids using composite hydrogel for on-chip assessment of immunomodulatory drug activity. Sabhachandani et al uses an integrated high-throughput microfluidic droplet docking array, which traps the spheroids on-chip to evaluate dynamic cell response to immunotherapy. The spheroids are continuously perfused with drugs, and the cell secretions are collected at routine intervals for proteomic analysis. This system has the advantage of facilitating dynamic analysis of cellular interaction, proliferation, and therapeutic efficacy via spatiotemporal monitoring.^62^ Other lymphoma-on-chip systems have been developed to date that attempt to study the complex interactions between tumor cells and the surrounding endothelium. In particular, using a simplistic fabrication process, Mannino et al developed a model in which a fully vascularized, perfusable, microfluid traverses a DLBCL tumor cell-laden hydrogel that successfully recapitulates the hallmark attributes and cellular interactions that occur within the DLBCL TME. Importantly, this model allows cellular signaling proteins and infused reagents to diffuse through the membrane before reaching the tumor niche.^63^ Finally, Bruce et al have engineered a 3D microfluidic cell culture platform with controlled fluidic shear stress, by encapsulating in a 3D collagen matrix primary human BM stromal cells, OB, and human ALL cells. Authors studied leukemia cell viability in response to a chemotherapeutic agent and showed chemotherapeutic drug sensitivity of leukemic cells in 3D triculture models to be decreased from the 2D models, suggesting that the BM environment has a protective effect.^64^

Despite these advantages, microfluidic systems may represent a limitation in the investigation of some pathophysiological processes, such as the effect of physiological shear forces on blood vessels or extravasation of cancer cells in complex tissue-like architectures; this aspect cannot be neglected in hematological cancers.

To overcome this limitation, several types of bioreactors have been introduced that provide critical biomechanical stimuli to cultured cells, including direct perfusion bioreactors, millifluidic bioreactors, rotating wall vessel bioreactors, and magnetic lift bioreactors (summarized in Figure 4). Moreover, these systems represent an important preclinical drug-testing platform, as bioreactors enable drug penetration and distribution in a non-homogeneous manner, such as in vivo. In particular, direct perfusion bioreactors enable direct medium perfusion through the pores of the scaffold millifluidic bioreactor. On the contrary, rotating wall vessel bioreactors provide a rotating dynamic system with lower shear stress, which is also able to mimic microgravity. Moreover, there are bioreactors that apply a selective, computer-controlled mechanical force such as dynamic compression, thus allowing for tissue formation under physiological loading conditions.^65^

Besides the above-mentioned systems, other (3D) human leukemic BM models have been proposed. Zippel et al generated a system in which perfusion and media exchange were ensured by a magnetic, parallelized culture system. Acute myeloid leukemia cell line KG-1a seeded with MSC revealed a greater resistance to chemotherapeutic treatment in the 3D magnetic hydrogel, compared with 2D culture. Furthermore, in the 3D tricultures with HSPC, MSC, and KG-1a, HSPC proliferation decreased while KG-1a cells remained unaffected posttreatment, imitating leukemic BM. Interestingly, a noninvasive metabolic profile allowed for continuous monitoring of the system. The results of this study highlight the importance of using biomimetic 3D platforms with proper media exchange and cocultures to create in vivo-like conditions and enable in vitro drug testing.^66^

We recently demonstrated the feasibility of recreating a surrogate 3D MM and CLL microenvironment able to reproduce the functional interactions of the native BM.^20,50,67^ We developed a robust technology based on the integrated use of cell-repopulated scaffolds (BM stromal cells) and the microgravity RCCS bioreactor. This system provides a balance between increased mass transfer and reduced shear stress, and this dynamic bioreactor generates optimal conditions for long-term ex vivo maintenance of tissue explants. Specifically, Belloni et al have shown that the model preserves, for extended time periods, the morphological and functional features of MM tissue components and their sensitivity to drugs.^50^ Of note, 2 distinct environments coexist in the bioreactor: 1 inside and 1 outside the scaffold. This aspect enabled us to evaluate CLL cell mobilization induced by specific drugs (in this case we used the BTK inhibitor ibrutinib), which is not possible in conventional 2D culture settings. This system also enabled us to study the phenotype of CLL cells in the 2 compartments, and we demonstrated that it mirrors the in vivo differences between tissue compartments.^20^

This model can be adapted to generate other relevant TME for hematological cancer by considering the use of additional cell types (including primary BMSC and EC for generation of vascularization) and other types of scaffolds. These findings indicate that 3D dynamic culture of reconstructed human BM in bioreactor may represent a useful platform for drug testing and for studying tumor-stroma molecular interactions.

There is also a growing interest in understanding and studying the (both healthy and leukemic) lymphocyte trafficking that likely contributes to the clinical course of many hematological cancers, among them CLL; however, this process is difficult to address in static in vitro culture. An example of a dynamic in vitro model in which CLL cells experience shear forces equivalent to those in capillary beds and are made to flow through capillary-like hollow fibers lined with EC has been published by Walsby et al.^68^ Thanks to this system, the authors could provide evidence for a novel, dynamic, and tractable in vitro model of lymphocyte migration, and confirm that CD49d is a critical regulator of this process in CLL.

We are currently implementing the use of bioreactors under millifluidic control coupled with both hydrogel or collagen-based scaffolds to achieve lymphoid tissue maturation and mimic CLL cell dissemination through the blood vessels in vitro.^69,70^

## CHALLENGES

As mentioned earlier in this review, the major obstacle to the generation of 3D models for blood cancers is the nonadherent nature of immune cells. The most widely used 3D methods for solid tumors are based on the self-assembling of malignant cells, eventually in the presence of stromal cells. Models that are not based on the use of an external matrix support rely on the adhesive abilities of cells and their tendency to stick together. For this reason, blood cancer 3D modeling is shifting its focus more and more toward scaffold-based models.^1^ Taking into account these considerations, along with the aforementioned evidence that highlights the importance of the microenvironment to the development and progression of neoplasms, it is becoming necessary to recapitulate TME complexity in vitro.^1,4,5^ Primary and secondary SLO are extremely complex structures from both the architectural and functional points of view. Those organs are characterized by a precise compartmentalization in specific functional niches, and a wide variety of cell populations at different stages of differentiation.^29,31–33^ It is similarly tricky to reproduce the structures of blood and lymphatic vessels and obtain vascularized tissues.

Having said that, the first and most demanding challenge is to find a suitable compromise, consistent with cell viability and proliferation, in terms of materials (eg, scaffold material, medium mix, and growth factors), techniques for 3D sample generation, culture timing, sample manipulation, and analysis. Scaffold-free models are characterized by a wide variety of techniques. Specifically to hematological cancers, multiple methods are often used together, also exploiting the use of hydrogels, for example, to create seeding platforms or to externally encapsulate spheroids, facilitating the stabilization of nonadherent cell aggregates.^38,41^ The scenario of scaffold-based models is more complicated, because it is more difficult to standardize cell seeding and it is also necessary to consider the physical properties of the scaffold, which can influence cell behavior that is also related to the type of tissue to be reproduced (eg, stiffness),^2,27^ or that can influence the sample analysis (eg, optical transparency of the matrix).^3^

The key approach in this context could be a stepwise increase in the complexity of the 3D culture, which is also necessary for another delicate and important aspect—the standardization and reproducibility of the models.^1,3^ The use of novel techniques such as 3D bioprinting and dynamic growth in bioreactors can be of great help.^10,57,60^ The bioprinting technique is helpful in several aspects, in particular to reproducing the architectural complexity of lymphoid tissues, because it is possible to depose predefined structures with different compositions.^57^ Since we are talking about blood cancers, the addition of a dynamic stimulus could enhance the model’s complexity, enabling a more physiological microenvironment and the potential to add malignant cells to a healthy in vitro 3D model of lymphoid tissue, with the possibility of comparing the physiological and neoplastic scenario.^60,61,71^

## FUTURE PERSPECTIVE AND FINAL CONSIDERATIONS

The impact that 3D cultures could have on biomedical research is becoming more and more evident, and technological advancement makes a wide selection of methods available, according to the specific needs. Our aim with this review was to give the reader the opportunity to explore the different 3D culture approaches already in use for hematological cancer treatment and, in parallel, to point out the complexity of the microenvironment that we need to recapitulate.

We reported some evidence previously published and our experience underlining the possible advantages of the use of 3D culture models as new preclinical models. However, this field is still at its infancy for hematological disorders and there are probably other important biological differences, advantages, and disadvantages that we must still consider and evaluate. Future research in this field should focus on validation of the established models and standardization of the readout protocols, particularly in the field of drug testing. The analytical part is crucial, as the protocols and reagents currently available for conventional 2D culture strategies largely do not apply to 3D cultures. Comparative studies between the 2D and 3D cultures are also essential to establishing the real additional value of the new strategies. In particular, we infer that the combination of macroscale perfusable systems, surface-modified synthetic scaffolds, and nondestructive real-time monitoring will provide advanced platforms for in vitro modeling of hematological cancers, with broad applications in basic research and preclinical drug development.

## AUTHOR CONTRIBUTIONS

DB and CS wrote the article.

## DISCLOSURES

The authors declare no conflicts of interest.

## SOURCES OF FUNDING

CS acknowledges financial support from EHA Advanced Research Grant 2020. Associazione Italiana per la Ricerca sul Cancro AIRC under IG 2018 - ID 21332 and Special Program on Metastatic Disease – 5 per mille 2119. Alternative Research & Development Foundation (ARDF) grant 2022.
